# Supplementary material for: Bronze Age non-elite mobility in Denmark examined through a new human-based bioavailable strontium isotope range
Source: PLoS One. 2026 Feb 6;21(2):e0341434. doi: 10.1371/journal.pone.0341434 (PMC12880727; doi:10.1371/journal.pone.0341434)
Supplement: S1 Appendix — This section describes the significance and relevance of the human-based strontium isotope range presented herein and compares it with other environmental proxy-based baselines that have been published for Denmark. (DOCX) [file pone.0341434.s002.docx]

**S1 Appendix. Significance of human-based bioavailable strontium isotope range and its comparison with modern proxy-based baselines**

Robert Frei

Citation numbers and references correspond to main text reference list.

**Significance of the human-based strontium isotope range**

The key study by [61] concluded that strontium isotope ratios in the otic capsule closely align with those in dental enamel from the same individuals, regardless of whether the remains were cremated or not. The petrous bone begins mineralizing before birth and continues to grow until about 2 years of age [61, 91, 92]. This finding underscores the petrous bone’s reliability in tracing childhood origins. Additionally, a more recent study by [77], which assessed the preservation of biogenic strontium isotope ratios in the otic capsule of unburnt petrous bones, supports Harvig and coworker’s [61] main conclusions. These studies collectively highlight the significance of the petrous bone in archaeological provenance investigations, particularly for inhumations and non-cremated remains, offering an additional source of information.

In comparison, the calcification of permanent maxillary and mandibular teeth starts after a few months after birth (incisors and canines) to between 1.5 to 9 years (premolars and molars) and completion of enamel formation is reached at around 1-2 years for the first molar, between 4-7 years for incisors, premolars and canines, and up to 7-16 years for second and third molars [91]. Hence, the strontium isotope signature of the petrous bone reflects an earlier childhood period compared to that represented by most of the tooth enamel samples.

In this view, and because pars petrosa bone and enamel are relatively inert toward diagenetic alterations of their strontium isotope signatures, the new human-based bioavailable strontium isotope range mirrors the childhood origins with respect to the bioavailable strontium intake of the many hundreds of individuals studied throughout the archaeological periods. It provides a conservative and statistically solid picture of mobility and migration of humans to Denmark from abroad.

**Changes of the human-based bioavailable strontium isotope signatures through time**

The combined dataset of compiled and newly generated values presented here enables an examination of potential trends in bioavailable ⁸⁷Sr/⁸⁶Sr signatures over archaeological time. S1 Figure 1 focuses on the enamel and pars petrosa samples used to define the ⁸⁷Sr/⁸⁶Sr range (represented by orange-filled symbols), specifically the range calculated after excluding outliers (shown as blue-filled symbols) identified using the median absolute deviation (MAD) method (see main text).

**S1 Figure 1. Temporal distribution diagram focusing on data of human-based strontium isotopes range-defining local individuals**. Temporal evolution diagram focusing on the human-based bioavailable strontium isotope range (indicated by a transparent yellow band across the diagram). Data points defining the bioavailable strontium isotope range are plotted with orange-filled symbols, blue symbols depict statistical outliers here interpreted as non-locals. A trendline through the baseline data is plotted with a dashed orange line. It has a slight positive slope that might hint at a progressively increasing average bioavailable signature in Denmark, potentially routed in an increased acidification of the glaciogenic topsoils and concomitant leaching of natural carbonate components through time. For details refer to text. The horizontal red dashed line marks the 20% limit of non-locals in the entire dataset. The x-axis depicts the sample numbers listed in S1 Table. Neo = Neolithic; BA = Bronze Age; IA = Iron Age; VA = Viking Age; MA = Medieval.

Although the trend is only weakly defined, there appears to be a slight overall increase in average ⁸⁷Sr/⁸⁶Sr values from the Neolithic to the Medieval period. This is illustrated by the positively sloped trendline (orange dashed line in S1 Figure 1), which extends from left to right across the plot. The trendline was calculated using the least squares method, which determines the line of best fit by minimizing the sum of the squared differences between observed data points and the corresponding predicted values.

Whether this subtle increase in bioavailable strontium isotope values through time is statistically significant remains uncertain. However, it may reflect an underlying trend in the bioavailable strontium isotope composition of the Danish landscape. If so, this shift could have influenced the average dietary strontium intake of past human populations.

Strontium (Sr) is derived from a wide variety of sources, but it is primarily consumed through food and water [93]. Several food groups make significant contributions to human dietary strontium intake. Plant-based foods typically contain higher strontium concentrations ([Sr] usually > ~1 to 100 mg/kg [94]; for example, [Sr] in plants from Denmark ranges from 2.5 to 89 mg/kg, with an average of ~20.2 mg/kg; [16]) than animal-based foods (e.g., [Sr] in cattle meat is much less than 1 mg/kg; [95]). As a result, plant-based foods generally have a greater influence on average strontium values in human remains [94]. Additionally, seafood—especially filter feeders such as shellfish—also contributes to dietary Sr intake.

Drinking water is another important source of strontium, especially in regions where natural Sr concentrations are elevated. In Denmark, for instance, strontium concentrations in surface waters typically range from 0.15 to 1.15 mg/L [17], primarily due to interactions between surface and groundwater and strontium-rich carbonate (i.e., limestone) components in the glaciogenic sediments [17, 35, 96, 97]. It has also been shown that food prepared with drinking water can contribute significantly to overall strontium intake [98].

Assuming that plants and drinking water were the primary dietary sources of strontium for ancient populations in Denmark, the human-based bioavailable strontium isotope range is likely to reflect the average composition of this combined intake. Plants acquire their Sr isotope signatures from the bioavailable strontium present in soils. In this context, we propose that the gradual increase in average ⁸⁷Sr/⁸⁶Sr ratios observed over time may be linked to progressive acid leaching of soils—particularly the leaching of natural carbonate components. This process, acting on Denmark’s glaciogenic soils, likely resulted in increasingly radiogenic bioavailable strontium, which would be taken up by plants and passed on to humans through their diet.

Such a scenario was recently proposed by [16] based on a nationwide survey of strontium isotope ratios in soil leachates and plants collected from pristine forest sites—areas minimally impacted by agricultural activity. Their findings indicate that present-day bioavailable Sr isotope signatures, as inferred from these modern proxies, may not reliably represent past conditions in Denmark or potentially in other regions of northern Europe covered by glaciogenic sediments from the last glacial period. Notably, the current range of ⁸⁷Sr/⁸⁶Sr values in soil leachates (0.708–0.715; [16]) is substantially broader and includes more radiogenic signatures than the earlier baseline defined for Danish surface waters (⁸⁷Sr/⁸⁶Sr = 0.7080–0.7112; [17]). The surface water-based baseline range is depicted in S1 Figure 2 where it is compared to the human-based bioavailable strontium isotope range defined herein.

**S1 Figure 2. Temporal distribution diagram comparing the human-based strontium isotope range with the surface water-based baseline**. Temporal evolution diagram focusing on the human-based bioavailable strontium isotope range (indicated by a transparent yellow band across the diagram). Data points defining the baseline are plotted with orange-filled symbols, blue symbols depict statistical outliers here interpreted as non-locals. The human-based bioavailable strontium isotope range is marked as a yellow transparent band across the diagram. Trendline (dashed orange line) as in S1 Figure 1. The baseline range defined by voluminous surface waters [17] is superimposed as a blue transparent band across the diagram. While both ranges significantly overlap, we note a slight shift of the human-based baseline to higher 87Sr/86Sr values relative to the surface water baseline. For details refer to text. The x-axis depicts the sample numbers listed in S1 Table. Neo = Neolithic; BA = Bronze Age; IA = Iron Age; VA = Viking Age; MA = Medieval.

The large dataset of human enamel and pars petrosa samples compiled in this study strongly supports the arguments of [16]. The human-derived bioavailable strontium isotope range of ⁸⁷Sr/⁸⁶Sr = 0.7089–0.7117, which likely reflects average dietary—and thus bioavailable—strontium isotope values over archaeological time, aligns only with the lower portion of the modern soil leachate and plant-based range reported by [16]; see S1 Figure 3 and S1 Figure 4).

**S1 Figure 3. Temporal distribution diagram comparing the human-based strontium isotope range with the soil leachate-based baseline**. Temporal evolution diagram focusing on the human-based bioavailable strontium isotope range (indicated by a transparent yellow band across the diagram). Data points within the range are plotted with orange-filled symbols, blue symbols depict statistical outliers here interpreted as non-locals. The human-based bioavailable strontium isotope range is marked as a yellow transparent band across the diagram. Trendline (dashed orange line) as in S1 Figure 1. The baseline range defined by modern soil leachates [16] is superimposed as a brown transparent band across the diagram. While both baseline ranges overlap, the soil-derived baseline is significantly broader and expanded towards higher 87Sr/86Sr values relative to the human-based range. This most likely is due to the progressive acid leaching of carbonate components in the glaciogenic soils over time. For details refer to text. The x-axis depicts the sample numbers listed in S1 Table. Neo = Neolithic; BA = Bronze Age; IA = Iron Age; VA = Viking Age; MA = Medieval.

**S1 Figure 4. Temporal distribution diagram comparing the human-based strontium isotope range with the plant-based baseline**. Temporal evolution diagram focusing on the human-based bioavailable strontium isotope range (indicated by a transparent yellow band across the diagram). Data points within the range’s upper and lower limits are plotted with orange-filled symbols, blue symbols depict statistical outliers here interpreted as non-locals. The human-based bioavailable strontium isotope range is marked as a yellow transparent band across the diagram. Trendline (dashed orange line) as in S1 Figure 1. The baseline range defined by modern plants [16] is superimposed as a green transparent band across the diagram. While both ranges overlap, the plant-based baseline is significantly broader and expanded towards higher ^87^Sr/^86^Sr values relative to the human-based range, similar to the soil-based baseline (S1 Figure 3). This most likely is due to the uptake of bioavailable strontium with elevated ^87^Sr/^86^Sr signatures from the respective soils that are affected by acid leaching over time. Both soil- and plant derived baselines do not adequately reflect the bioavailable signatures in the past and cannot therefore be used in mobility studies of ancient humans and animals. For details refer to text. The x-axis depicts the sample numbers listed in S1 Table. Neo = Neolithic; BA = Bronze Age; IA = Iron Age; VA = Viking Age; MA = Medieval.

In conclusion, the slight increase observed in average ⁸⁷Sr/⁸⁶Sr signatures over time could plausibly result from a rise in the strontium isotope values of plant-based foods consumed by humans—an effect expected from the progressive acid leaching of surface soils. Alternatively, it may reflect a concurrent increase in the ⁸⁷Sr/⁸⁶Sr ratio of surface water used for drinking, or a combination of both factors.

**Comparison of the human-based bioavailable strontium isotope range with other proxy baselines**

Interestingly, the human-based bioavailable strontium isotope range presented in this study (⁸⁷Sr/⁸⁶Sr = 0.7089 to 0.7117) is slightly shifted toward more radiogenic values compared to the surface water–based baseline proposed by [17], which spans ⁸⁷Sr/⁸⁶Sr = 0.7080 to 0.7112 (see S1 Figure 2). Although the water-based baseline still accounts for approximately 91.3% of the human-based data and identifies 20.5% of the entire human dataset as non-local, the observed offset toward lower ⁸⁷Sr/⁸⁶Sr values in surface waters warrants further consideration.

One proposed explanation, originally put forward by [33], suggests that modern Danish surface waters may be contaminated by strontium derived from agricultural liming—a widespread practice implemented over the past century to increase soil alkalinity and improve crop yields. However, this hypothesis has been robustly challenged by subsequent studies [35, 99], which demonstrated that strontium released from agricultural lime is largely retained in the uppermost ~60 cm of limed soil profiles due to strong adsorption onto organic material. These findings indicate that the mobility of lime-derived Sr into the vadose zone—and ultimately into surface waters—is minimal. In fact, [99] showed that the majority of Sr added through decades of liming remains trapped in the topsoil to this day.

This topic has sparked considerable debate (e.g., [33, 35, 96, 99-102]). While a detailed engagement with this controversy lies beyond the scope of the present study, the most recent evidence suggests that strontium from agricultural lime does not significantly contribute to surface runoff.

Accordingly, we do not interpret the shift toward less radiogenic ⁸⁷Sr/⁸⁶Sr values in the surface water baseline as a result of modern agricultural contamination. Rather, we favor an alternative explanation, as discussed in the previous section: the human-based bioavailable strontium isotope range may be slightly elevated in radiogenic strontium due to dietary intake of plant-based foods that reflect progressively leached glaciogenic soils. Over time, acid leaching of these soils likely increased the radiogenic component of bioavailable Sr, leading to elevated ⁸⁷Sr/⁸⁶Sr signatures in plants—and consequently in the human strontium isotope record.

Following the recommendations of [33] and [102] to focus on measuring bioavailable Sr isotope signatures from modern, "pristine" (i.e., agriculturally uncontaminated) forest soils, [16] conducted a nationwide study. This study presented over 160 soil-leachate and more than 160 plant analyses from such pristine sites. The baseline ranges reported—⁸⁷Sr/⁸⁶Sr = 0.7118 ± 0.0037 (2σ; n = 161) for soil leachates and 0.7115 ± 0.0025 (2σ; n = 162) for plants—are notably broader and extend toward higher ⁸⁷Sr/⁸⁶Sr values than the human-based bioavailable strontium isotope range advocated in the present study (see S1 Figure 3 and S1 Figure 4).

It is evident that modern proxies from uncontaminated Danish sites (e.g., unaffected by agricultural liming) do not accurately represent the human-based bioavailable strontium isotope range, which better reflects actual dietary Sr intake. Frei and coworkers [16] have already addressed this issue in detail, strongly arguing against the use of these modern proxy baselines for provenance studies of ancient humans and animals in Denmark. Such proxies do not appear to reliably reflect the past bioavailable Sr landscape.

When applied, the modern soil-leachate and plant baselines would classify only 15 and 23 out of 628 human samples, respectively, as non-local—corresponding to just 2.4% and 3.7%. These figures fall well below the 5–30% non-local expectation for even relatively sedentary ancient populations (as discussed above) and stand in stark contrast to the approximately 14% non-local rate identified using the new human-based bioavailable strontium isotope range.

Over a decade ago, [37] published strontium isotope data from modern mice (extracted from owl pellets collected across Denmark) and archaeological fauna, comparing these values to the median ⁸⁷Sr/⁸⁶Sr signatures of human enamel from various Danish archaeological sites. These sites spanned the Neolithic, Mesolithic, Iron Age, Viking Age, and Medieval periods. The faunal samples reported in that study ranged from ⁸⁷Sr/⁸⁶Sr = 0.70717 to 0.71185, with an average of 0.70919, while median values for human enamel populations ranged from 0.7086 to 0.7110, with an average of 0.7098.

Importantly, the faunal-based ⁸⁷Sr/⁸⁶Sr range encompasses 100% of the human-based bioavailable strontium isotope range and identifies approximately 10.9% of the total human dataset as non-local. Furthermore, the range of median enamel values from the 17 archaeological sites presented by [37] aligns closely with the human-based bioavailable strontium isotope range proposed in the present study (⁸⁷Sr/⁸⁶Sr = 0.71031 ± 0.00140; 2σ; n = 564). These earlier data thus reinforce the validity of the new baseline as a reliable representation of the average dietary strontium isotope composition of past populations in Denmark.

The vast majority of faunal samples from [37] are consistent with the human-based bioavailable strontium isotope range. However, a few outliers—mouse bones from owl pellets collected in parts of Jutland—exhibit unusually low ⁸⁷Sr/⁸⁶Sr values (<0.708), falling outside the defined human-based range. While this study does not focus on these exceptions, we speculate that such low values may result from bioavailable Sr derived from volcanic ash layers, particularly those exposed in the western Limfjorden area of northern Jutland. These ash layers have reported ⁸⁷Sr/⁸⁶Sr values ranging from ~0.7035 to 0.7079 [103].

The Limfjorden region is known to exhibit relatively lower ⁸⁷Sr/⁸⁶Sr values in surface waters, as shown in kriged maps published by [17] and [96]. Surprisingly, however, these lower ratios are not consistently mirrored in human enamel signatures from the same region, nor are they visible in the kriged maps of soil-leachate - and pant strontium isotope signatures published by [96]. Despite the inclusion of dozens of samples from multiple archaeological sites in Limfjorden (see Fig. 1 and S1 Table), human data across all time periods rarely reflect these expected lower values.

Only a handful of individuals from Limfjorden show ⁸⁷Sr/⁸⁶Sr values below the lower human-based strontium isotope range limit of 0.7089. These include one individual from Site 85 (on the island of Fur, where volcanic ash layers are exposed), another from Site 96 (see Fig. 1), and samples Lim-ht-066*, Lim-ht-068, and Lim-ht-192 from [36], encompassed by the rectangular area “A” in Figure 1 of the current study. While these outliers are noteworthy, a full explanation lies beyond the scope of this contribution and should be addressed in future, site-specific investigations.
