# Supplementary material for: Bronze Age non-elite mobility in Denmark examined through a new human-based bioavailable strontium isotope range
Source: PLoS One. 2026 Feb 6;21(2):e0341434. doi: 10.1371/journal.pone.0341434 (PMC12880727; doi:10.1371/journal.pone.0341434)
Supplement: Attachment — Submitted filename: Frei at all editorial supplement.pdf [file pone.0341434.s007.pdf]

Sr8786	N.Freq
0,70883	2
0,70913	2
0,70931	2
0,70934	2
0,70941	2
0,70949	2
0,70971	2
0,70978	2
0,70980	2
0,70986	2
0,70988	2
0,70990	2
0,70999	2
0,71002	2
0,71004	2
0,71005	2
0,71011	2
0,71012	2
0,71016	2
0,71021	2
0,71022	2
0,71029	2
0,71032	2
0,71034	2
0,71039	2
0,71041	2
0,71042	2
0,71044	2
0,71053	2
0,71054	2
0,71056	2
0,71060	2
0,71070	2
0,71078	2
0,71087	2
0,71088	2
0,71096	2
0,71099	2
0,71100	2
0,71105	2
0,71110	2
0,71111	2
0,71123	2
0,71127	2
0,71129	2
0,71134	2
0,71136	2
0,71137	2
0,71150	2

0,71166	2
0,71167	2
0,71187	2
0,71195	2
0,71199	2
0,71203	2
0,71260	2
0,70920	3
0,70930	3
0,70959	3
0,70985	3
0,70988	3
0,70989	3
0,70996	3
0,70998	3
0,71000	3
0,71003	3
0,71013	3
0,71018	3
0,71019	3
0,71023	3
0,71025	3
0,71040	3
0,71050	3
0,71061	3
0,71065	3
0,71073	3
0,71076	3
0,71094	3
0,71095	3
0,71140	3
0,71180	3
0,71190	3
0,70937	4
0,70944	4
0,70970	4
0,71028	4
0,71030	4
0,71082	4
0,71085	4
0,71090	4
0,70940	5
0,71074	5
0,71080	5
0,71084	5
0,71010	6
0,71020	6

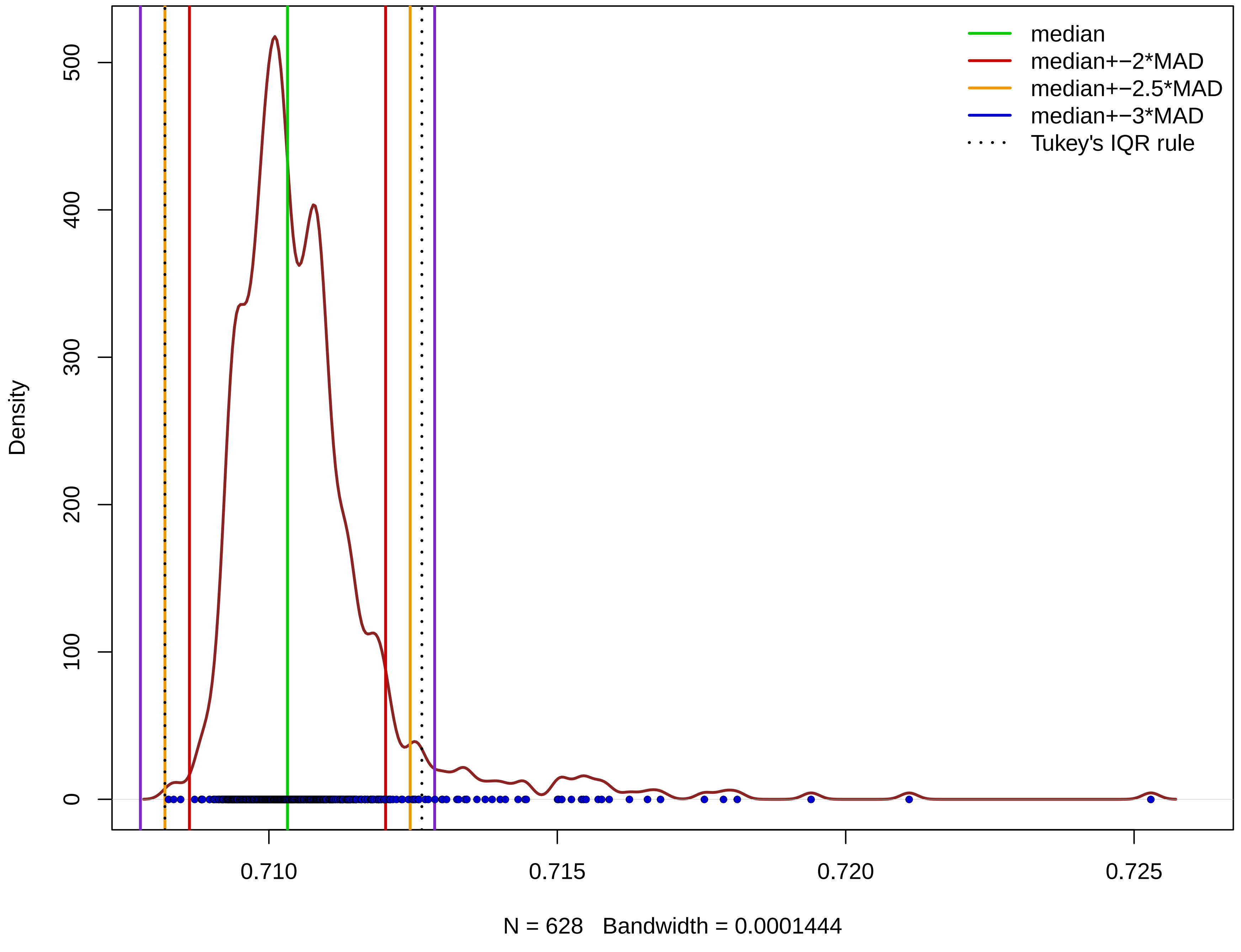

median
median+−2*MAD
median+−2.5*MAD
median+−3*MAD
Tukey's IQR rule
Density
0
100
200
300
400
500
0.710
0.715
0.720
0.725
N = 628   Bandwidth = 0.0001444
