# Supplementary material for: Bronze Age non-elite mobility in Denmark examined through a new human-based bioavailable strontium isotope range
Source: PLoS One. 2026 Feb 6;21(2):e0341434. doi: 10.1371/journal.pone.0341434 (PMC12880727; doi:10.1371/journal.pone.0341434)
Supplement: Attachment — Submitted filename: Response to Reviewers.docx [file pone.0341434.s008.docx]

Response to reviewers

PONE-D-25-53013

Original title: A new human-based strontium isotope baseline for Denmark and its application to Bronze Age non-elite mobility

New revised title: Bronze Age non-elite mobility in Denmark examined through a new human-based bioavailable strontium isotope range

We sincerely appreciate the detailed and constructive comments provided by both reviewers and the academic editor, Dr. Luca Bondioli. We are pleased that the reviewers agree in that the compiled and new supplemented human dataset are a valuable resource for future archaeological provenance studies in Denmark. While reviewer 1# has only minor comments, we are pleased to also read the conclusion of reviewer #2 who writes that “the dataset presented is of great value to the archaeological community”. We are therefore grateful that both reviewers see the value of this study and contribution to the research community. At the same time, we acknowledge that several aspects of our original manuscript were not presented with sufficient clarity, which contributed to some misunderstandings. We have now clarified these points in the revised version.

Below, we outline the revisions we have made in response to the reviewers’ thoughtful comments. We address each point individually in a point-by-point manner, with all changes highlighted in blue.

The manuscript by Frei et al. presents a rich database aimed at creating a baseline specific to Denmark for the 87/86 strontium isotope ratio.

Both reviewers welcomed the manuscript but also raised some issues and topics for discussion that need to be addressed by the authors in order to make the manuscript publishable in Plos One.

In particular, reviewer 2 raised some doubts about the basis of the analysis itself. As far as I am concerned, I believe that the manuscript should take into account the reviewers' criticisms, considering them as constructive contributions (even beyond the form). The criticisms should be integrated into the text, also showing possible alternative scenarios with a less assertive and more critical approach. Currently, the manuscript has a dual hybrid dimension, the first being a review paper (without the necessary insights), and the second one being a definition of the baseline (without an effective critical approach).

We would like to clarify that our manuscript is not intended to be read as a review paper. Rather, its primary aim is to present—for the first time—strontium isotope data from non-elite individuals from the Nordic Bronze Age excavated within present-day Denmark. Although this group of individuals is crucial for understanding the socio-dynamics of this formative European period, it has remained largely “invisible” in the archaeological record of the Nordic Bronze Age. Our study provides the first systematic attempt to address this gap in the region of present-day Denmark.

Second, to contextualize these new data, we compiled the first comprehensive database of published strontium isotope measurements from archaeological human remains (tooth enamel and pars petrosa bone) across present-day Denmark (excluding Bornholm). This dataset provides a valuable comparative reference for Sr isotope studies. Based on this compilation, we propose a statistically derived human-based bioavailable strontium isotope range representative for this region. We only propose this approach for this region because:

1. **Denmark’s relatively homogeneous, glaciogenic surface geology**, which was also exemplified by the resulting relatively narrow surface-water baseline published by Frei and Frei (2011), which suggests that due to this unique geological framework, substantial large-scale variability in bioavailable Sr isotopes are not expected; and
2. **the substantial corpus of accumulated, but previously scattered, published human Sr isotope data** (summarized in Table S1 with full source references) is now sufficient to support robust statistical analyses of past bioavailable ranges. This combination of relatively homogeneous, glaciogenic surface geology and extensive data coverage creates a unique situation in which a national-level human-based range can be meaningfully defined. We explicitly acknowledge that this approach is only valid under such geological conditions and is not transferable to regions with more heterogeneous geological settings.

We also recognize that our earlier use of the term “baseline” may have caused confusion regarding the nature of this human-based range. We have therefore removed the word “baseline” and now refer solely to a “range.”

Furthermore, unlike traditionally defined baselines or isoscapes, our dataset is not spatially geo-referenced in a way that reflects local environmental conditions at specific geographic locations. In this regard, we fully agree with Reviewer #2. Consequently, our archaeological dataset cannot be used to construct an isoscape for Denmark (e.g., through kriging), and our proposed national range is not intended for assessing intra-Denmark mobility patterns.

In our revised manuscript we have provided interpretations of the new data from the non-elite Bronze Age individuals, in relation to the two previously publblished baselines for Denmark. Furthermore, we compare these interpretations with how the related to the comprehensive dataset in terms of the range. This allows us to distinguish individuals who are “local” (i.e., originating from the region of present-day Denmark excluding the island of Bornholm) from those who are “non-local”. We present the different possible interpretations depending on which previous baselines are used, and how these interpretations compare to the range calculated from the human-based database.

In other words, to avoid further misunderstanding, we have adopted Reviewer #2’s suggestion and refrain from referring to this range as a “baseline.” We instead describe it as a **statistically well-defined, human-based bioavailable strontium isotope range**, which can be used as an additional reference material alongside existing proxy-based baselines such as the surface-water baseline of Frei and Frei (2011) and the fauna/human-based reference range of Frei and Price (2012). The Supplementary Information also provides a detailed comparison of our human-based range with previously established baselines derived from surface water, soil leachates, and plants.

Finally, we emphasize that the presentation of this human-based range is only one component of the study. The principal objective remains the investigation of mobility (or lack thereof) among non-elite Bronze Age individuals in present-day Denmark—an aspect that has never before been studied in this region.

Beyond these general considerations, I must add a series of notes:

1. The database in Table S1 must also include a column indicating whether the individuals were cremated or inhumed.

We have added two additional columns specifying “non-cremated” (which include inhumations, bog and midden finds, and finds in gallery graves and sacrificial wells) and “cremated”.

2. The database contains values with four or five decimal places: where possible, it would be advisable to standardise the values as much as possible to the fifth decimal place.

S1 Table contains the data as they were published in the respective literature. Some publications only state 4 digits in the 87Sr/86Sr ratios, some do not report errors. Where possible, we now report data to 5 significant digits. This is not possible for cases where data are reported to 4 digits only in the respective source literature cited.

3. I have carried out a quick analysis of the data in Table 6: as can be seen in the attached PDF file, 40.4% of the isotopic ratio values are present in at least two individuals, up to a maximum of six individuals, all with a value of 0.7102, and six with a value of 0.7101. Twenty-three per cent of the duplicates have isotope ratio values to four decimal places, but the remaining 77% are values to five decimal places. Although a certain percentage of duplicate data may be “reasonable” in an analysis of this type, I feel that these percentages are excessive and I invite the authors to further check Table S1. Finally, I think that this situation, if confirmed, should be discussed in the article and justified.

We appreciate the effort in inspecting the data. As mentioned above under point 2, we report data as they were published in the respective literature. Hence, individuals with isotopic values reported only with 4 digits of the 87Sr/86Sr ratios (i.e., ratios that were rounded to the fourth digit) might appear as duplicates, but in reality, they are pseudo-duplicates. However, we are restricted to the reporting of the data as appearing in the respective literature.

4. In my opinion, the use of MAD should be better justified with more literature. Furthermore, the choice to use a multiplier of 2 to define the range of “local” individuals should be better justified (also because the bibliography cited by the authors suggests a value of 2.5). I have taken the liberty of attaching a graph showing the different ranges resulting from different multiplier values. Interestingly, the value of 2.5 seems to be close to the value of Tukey's method, which is the most widely used in the literature for defining outliers.

We appreciate the comment regarding the statistical method used and the effort in illustrating how different MAD parameters, as well as how the Tukey method, affect the results. While we have considered Tukey’s method, we have chosen a MAD approach to delineate outliers in our dataset, as the distribution is very narrow around the median (high kurtosis) with a strong, but flat, positive tail (positive skewness). For this reason, we applied a multiplier factor of 2 in our MAD approach. As described by Leys et al. (2013), this factor is considered “poorly conservative,” yet it effectively captures and delineates outliers in the flat tail as well as slightly asymmetric portions of the main data distribution—particularly elevated ⁸⁷Sr/⁸⁶Sr values—without overestimating outliers. The effectiveness of this approach is reflected in the very low skewness and kurtosis values reported in Table 2, indicating a well-defined Gaussian distribution after outlier removal.

In our study, where we assume that the majority of humans are of local origin (i.e., within present-day Denmark). In this context, the median is an ideal reference. By contrast, Tukey’s method, which uses interquartile ranges, is less robust and could underestimate the number of outliers—particularly for data in the slightly asymmetric portion of the central distribution that would not be classified as outliers using Tukey’s approach.

While a detailed discussion of the advantages and disadvantages of different statistical methods is beyond the main scope of our manuscript, we have added a paragraph in the section entitled “The ⁸⁷Sr/⁸⁶Sr database of archaeological human remains” that elaborates on the reasoning of our choice. In this paragraph, we also added the reference Lightfoot and O’Connell (2016), who recommend using both, MAD and Tukey’s IQR, as a robust approach for identifying migrants in archaeological population samples, based on a global survey of oxygen isotopes in ancient human tooth enamel and bone bioapatite.

PLOS ONE

Journal Requirements:

When submitting your revision, we need you to address these additional requirements.

1. Please ensure that your manuscript meets PLOS ONE's style requirements, including those for file naming. The PLOS ONE style templates can be found at

https://journals.plos.org/plosone/s/file?id=wjVg/PLOSOne_formatting_sample_main_body.pdf and

https://journals.plos.org/plosone/s/file?id=ba62/PLOSOne_formatting_sample_title_authors_affiliations.pdf

2. In your manuscript, please provide additional information regarding the specimens used in your study. Ensure that you have reported human remain specimen numbers and complete repository information, including museum name and geographic location.

All but the samples from the two sites from which new data is reported have been published in the literature with the necessary sample information. We found some additional sample specifications for some of the compiled data in the respective literature and added some of the museum numbers lacking in S1 Table where possible.

With respect to the specimens from the new data generated in this study all details are reported herein including the museum numbers, and geographic location.

If permits were required, please ensure that you have provided details for all permits that were obtained, including the full name of the issuing authority, and add the following statement:

'All necessary permits were obtained for the described study, which complied with all relevant regulations.'

If no permits were required, please include the following statement:

'No permits were required for the described study, which complied with all relevant regulations.'

We have added this disclosure to the acknowledgments.

3. Thank you for stating the following financial disclosure:

This study was made possible through the funding to KMF provided by the Carlsberg Foundation “Semper Ardens” advance research grant CF18-0005 for which we are very grateful.

https://www.carlsbergfondet.dk/en

We added this disclosure to the acknowledgments.

Please state what role the funders took in the study. If the funders had no role, please state: "The funders had no role in study design, data collection and analysis, decision to publish, or preparation of the manuscript."

We have added this statement to the cover letter.

If this statement is not correct you must amend it as needed.

Please include this amended Role of Funder statement in your cover letter; we will change the online submission form on your behalf.

4. We note that Figure 1 in your submission contain map images which may be copyrighted. All PLOS content is published under the Creative Commons Attribution License (CC BY 4.0), which means that the manuscript, images, and Supporting Information files will be freely available online, and any third party is permitted to access, download, copy, distribute, and use these materials in any way, even commercially, with proper attribution. For these reasons, we cannot publish previously copyrighted maps or satellite images created using proprietary data, such as Google software (Google Maps, Street View, and Earth). For more information, see our copyright guidelines: http://journals.plos.org/plosone/s/licenses-and-copyright.

We require you to either (a) present written permission from the copyright holder to publish these figures specifically under the CC BY 4.0 license, or (b) remove the figures from your submission:

We have purchased the background map image from iStock and redrafted Figure 1 with this background image. We provide written permission of iStock to publish this map image under the CC BY license in a separate uploaded "Other" file with our submission of the revised manuscript version. We also correctly refer to the source and credit in the respective figure caption.

a. You may seek permission from the original copyright holder of Figure 1 to publish the content specifically under the CC BY 4.0 license.

We recommend that you contact the original copyright holder with the Content Permission Form (http://journals.plos.org/plosone/s/file?id=7c09/content-permission-form.pdf) and the following text:

“I request permission for the open-access journal PLOS ONE to publish XXX under the Creative Commons Attribution License (CCAL) CC BY 4.0 (http://creativecommons.org/licenses/by/4.0/). Please be aware that this license allows unrestricted use and distribution, even commercially, by third parties. Please reply and provide explicit written permission to publish XXX under a CC BY license and complete the attached form.”

Please upload the completed Content Permission Form or other proof of granted permissions as an ""Other"" file with your submission.

In the figure caption of the copyrighted figure, please include the following text: “Reprinted from [ref] under a CC BY license, with permission from [name of publisher], original copyright [original copyright year].”

We added this text in caption of Figure 1.

b. If you are unable to obtain permission from the original copyright holder to publish these figures under the CC BY 4.0 license or if the copyright holder’s requirements are incompatible with the CC BY 4.0 license, please either i) remove the figure or ii) supply a replacement figure that complies with the CC BY 4.0 license. Please check copyright information on all replacement figures and update the figure caption with source information. If applicable, please specify in the figure caption text when a figure is similar but not identical to the original image and is therefore for illustrative purposes only.

The following resources for replacing copyrighted map figures may be helpful:

USGS National Map Viewer (public domain): http://viewer.nationalmap.gov/viewer/

The Gateway to Astronaut Photography of Earth (public domain): http://eol.jsc.nasa.gov/sseop/clickmap/

Maps at the CIA (public domain): https://www.cia.gov/library/publications/the-world-factbook/index.html and https://www.cia.gov/library/publications/cia-maps-publications/index.html

NASA Earth Observatory (public domain): http://earthobservatory.nasa.gov/

Landsat: http://landsat.visibleearth.nasa.gov/

USGS EROS (Earth Resources Observatory and Science (EROS) Center) (public domain): http://eros.usgs.gov/#

Natural Earth (public domain): http://www.naturalearthdata.com/

5. If the reviewer comments include a recommendation to cite specific previously published works, please review and evaluate these publications to determine whether they are relevant and should be cited. There is no requirement to cite these works unless the editor has indicated otherwise.

No recommendation to cite specific previously published works was made by the reviewers.

[Note: HTML markup is below. Please do not edit.]

Reviewers' comments:

Reviewer's Responses to Questions

Comments to the Author

1. Is the manuscript technically sound, and do the data support the conclusions?

The manuscript must describe a technically sound piece of scientific research with data that supports the conclusions. Experiments must have been conducted rigorously, with appropriate controls, replication, and sample sizes. The conclusions must be drawn appropriately based on the data presented.

Reviewer #1: Yes

Reviewer #2: Partly

________________________________________

2. Has the statistical analysis been performed appropriately and rigorously?

Reviewer #1: N/A

Reviewer #2: No

________________________________________

3. Have the authors made all data underlying the findings in their manuscript fully available?

The PLOS Data policy requires authors to make all data underlying the findings described in their manuscript fully available without restriction, with rare exception (please refer to the Data Availability Statement in the manuscript PDF file). The data should be provided as part of the manuscript or its supporting information, or deposited to a public repository. For example, in addition to summary statistics, the data points behind means, medians and variance measures should be available. If there are restrictions on publicly sharing data—e.g. participant privacy or use of data from a third party—those must be specified.

Reviewer #1: Yes

Reviewer #2: Yes

________________________________________

4. Is the manuscript presented in an intelligible fashion and written in standard English?

PLOS ONE does not copyedit accepted manuscripts, so the language in submitted articles must be clear, correct, and unambiguous. Any typographical or grammatical errors should be corrected at revision, so please note any specific errors here.

Reviewer #1: Yes

Reviewer #2: Yes

________________________________________

5. Review Comments to the Author

Please use the space provided to explain your answers to the questions above. You may also include additional comments for the author, including concerns about dual publication, research ethics, or publication ethics. (Please upload your review as an attachment if it exceeds 20,000 characters)

Reviewer #1: The paper of Frei et al reviews the human Sr isotope data from Denmark and uses this dataset as a way to detect local and non-local individuals among two archaeological sites. I have only minor comments that follow.

I do believe lines from 45 to 55 should be moved at the end of the introduction.

We have restructured the introduction chapter and, in this connection, also moved this section to the end of it.

The introduction is a bit weirdly structured: from the title and the abstract a reader expects to find a rationale behind the use of human samples to reconstruct local bioavailable Sr and then the application of this to a case study (non-elite individuals.). Overall, I think the authors should adjust the introduction following the abstract flow.

We recognize that the structure of the original draft may not have been presented with adequate clarity, and we have consequently reorganized and revised it to improve readability.

Figure 1: I think a (simplified) geological map would be important to show.

The bioavailable strontium in Denmark is essentially constrained to soils (predominantly luvisols and podsols) developed in glaciogenic tillites which overly the Cretaceous-Tertiary sediments. The latter, as shown by the study of Frei et al. (2022), has only minimal impact on the modern bioavailable strontium isotope compositions, and consequently modern surface water-, soil leachate – and plant- based proxies reveal a rather homogeneous range of 87Sr/86Sr over entire Denmark (cf. Frei and Frei, 2011; Frei et al., 2022). This circumstance makes Denmark unique as it allows for the construction of country-wide respective baselines. We therefore prefer to restrain from providing a geological map here as we think it will create confusion, and instead we refer to the soil map published by Frei et al. (2022) for completeness. A paragraph summarizing this has been added at the beginning of the section entitled “The ⁸⁷Sr/⁸⁶Sr database of archaeological human remains”.

Line 128: “we compile 513 previously published strontium isotope” please indicate here from how many sites.

We have added that the samples come from 159 sites.

Table 2: please use a consistent and reasonable number of digits.

We have reduced the number of digits to a reasonable degree in this table.

Table S1: why for some samples the 2SE is not reported?

We report the data as they appear in the respectively cited literature. Quite some publications unfortunately do not report the errors of the 87Sr/86Sr analyses.

Line 382: the result section starts from the case study and then jump to the Denmark baseline definition. I describing the baseline should come before.

As it was the case in the introduction, we recognize that the structure of the original draft may not have been presented with adequate clarity, and we have consequently reorganized and revised it to improve readability also within the results section.

Line 427: the authors used the entire dataset for the MAD approach. This means that the ‘baseline’ they calculate and refer to encompasses all of Denmark. Consequently, individuals identified as ‘non-local’ are likely those originating from outside the current boundaries of Denmark. While this approach is valid, the authors should clarify this methodological choice and provide a rationale for it. In contrast, my preferred approach would have been to calculate separate MAD values for each area or sub-region within Denmark. In addition, I think it would be important to clarify, maybe in the method section, what the authors mean by ‘non-local’.

We appreciate Reviewer #1’s acknowledgement that our approach—using the entire dataset to define a country-wide reference range—is valid within the specific geological context of Denmark, despite the reviewer’s preference for subdividing the dataset into regional units. Following this comment, we have expanded our explanation of the rationale behind this methodological choice, particularly the geological considerations, and we have clarified our use of the terms “local” and “non-local”.

Line 427-437: I think this part pertains the method section

We fully appreciate this suggestion; however, because our restructuring of the manuscript, we have integrated the relevant material into a subsection entitled “The ⁸⁷Sr/⁸⁶Sr database of archaeological human remains” within the Results section.

Reviewer #2: General Assessment

This manuscript presents an ambitious and valuable dataset, compiling 628 human 87Sr/86Sr values from archaeological sites across Denmark. This database, in itself, is a significant contribution and will serve as an important reference for future work in the region.

We appreciate the reviewer’s perspective of the significance of the dataset with respect to its value and use in future provenance work within Denmark

However, the manuscript's central methodological premise and its primary conclusion—that a single, nationwide "baseline" (0.7089–0.7117) can be derived from this national dataset using simple statistics and then be applied to provenance individuals—is fundamentally flawed.

We acknowledge that the term “baseline” we used here might cause misunderstandings. We respect this comment, and we now refrain from using the term “baseline” and instead refer to a “human-based bioavailable strontium isotope range”.

At the same time, we acknowledge that several aspects of our original manuscript were not expressed with sufficient clarity, which led to some misunderstandings.

However, as mentioned by reviewer 1# a nationwide “range” is a valid approach within the specific geological context of Denmark. Denmark’s geological setting is indeed unique: the entire country (with the exception of Bornholm) is covered by glacial sediments that are, to varying degrees, carbonate-bearing—carbonates being the dominant source of bioavailable Sr. As demonstrated in our previous studies (Frei and Frei 2011; Frei et al. 2022), contemporary bioavailable strontium isotope signatures—measured in surface waters (rivers and lakes), soil leachates, and plants—show only minor variation and are geographically homogeneous. This relative homogeneity is clearly illustrated in the kriged maps published in these earlier works.

We acknowledge that this distinctive situation differs markedly from countries where exposed basement rocks strongly influence environmental strontium and create greater spatial variability. In the Danish context, the geological uniformity supports the use of a country-wide human-based Sr isotope range to distinguish between “local” and “non-local” individuals. Here, “local” refers to individuals who spent their childhood and adolescence within the boundaries of present-day Denmark, while “non-local” refers to those who spent these formative years outside Denmark and later migrated into the region.

We have now clarified these points in detail in the revised manuscript.

The authors correctly criticize the limitations of environmental isoscapes (e.g., those based on modern water or soil), but they fail to demonstrate that their proposed alternative is a more suitable or accurate tool for provenancing. In fact, the proposed method ignores the single most important factor in strontium-based mobility studies: spatial variability.

We fully understand the reviewer’s point. And we realize that our original text was unclear. We don’t think that there is “one size fits all” in terms of how to create baselines. And we realize that our use of the word “baseline” in the original draft created unnecessary assumptions. Our intention was never to use the compiled human dataset to construct isoscapes—geolocation-based isotope distribution maps—in the traditional sense. The human data belong to a different category and do not reliably capture geographic variation at specific locations, because individual mobility introduces a potential “non-local” component at each site.

With respect to the spatial variability, we realize that our original manuscript was not clear enough in stating that our approach is only relevant to relatively unique - geologically speaking- homogeneous settings like the one in Denmark (excluding the island of Bornholm). We have therefore added some additional text, and also added a sentence in the abstract regarding that Denmark’s unique geological setting, i.e. that it is covered by relatively homogeneous glacial tillites which dominate the bioavailable strontium signature today (as reflected in surface waters, soil leachates, and plants), speaks for a limited spatial variability of Sr isotope signatures within this region. Furthermore, we emphasize that the underlying Cretaceous–Tertiary sediments are largely shielded by this homogeneous tillites, preventing the underlying geology from substantially influencing surface bioavailable strontium. Consequently, in this particular setting, it is possible to define a country-wide bioavailable strontium isotope range. Additionally, we also agree with the reviewer that this range cannot resolve movement within Denmark. Accordingly, the terms “local” and “non-local” refer to individuals who spent their childhood and adolescence within present-day Denmark (excluding the island of Bornholm) versus those who migrated to Denmark from elsewhere during the same life stages. So, in our opinion this situation unfortunately prevents reliable movement differentiation within this region.

We have now included a more detailed paragraph explaining this reasoning in the section entitled “The ⁸⁷Sr/⁸⁶Sr database of archaeological human remains” and we hope that it is now clear that we mean only in this region where this approach is possible and makes sense.

Major Points for Revision

1. The Methodological Fallacy of a Single National Baseline

The core flaw of this paper is the assumption that a single 87Sr/86Sr range can represent "local" for the entire nation of Denmark. The authors' own sources and the wider literature (e.g., Frei & Frei, 2011) show that bioavailable strontium is spatially variable across Denmark due to its complex glacial geology.

We understand that it is not often that country-wide ranges are reported, but this is due to the unique circumstances within this region, therefore the Frei & Frei (2011) article also proposes a nationwide baseline. While the distribution of glaciogenic sediments from different glacier advance and retreat stages within Denmark can indeed be mapped, the isotope signature of bioavailable strontium—extracted from surface soils, plants, and, to some extent, surface waters—is relatively homogeneous and exhibits little spatial variation. This is clearly illustrated by the kriged isoscape maps in Frei et al. (2022).

As noted in our previous response, we have now included a detailed passage explaining the unique circumstances in Denmark, which helps to explain why in the particular case of Denmark, a country-wide range is reported here.

The authors' approach conflates two different concepts: "local" (an individual originating from the specific geological catchment of the burial site) and "Danish" (an individual originating from anywhere within the modern political borders of Denmark).

We fully agree with the reviewer that a definition of the term “local” would help the reader to understand what we mean with it. In our revised draft we have therefore included the interpretation of the non-elite Bronze Age individuals in relation to the established baselines (i.e. Frei & Frei, 2011, and Frei & Price, 2012), and in each case we explain what is meant when an individual is interpreted as “local” or “non-local”. We do the same when we compare to the human-based bioavailable strontium isotope range calculated in this study.

By "flattening" all of Denmark's diverse geological signatures into a single statistical range, the authors' baseline becomes (a) too broad to detect inter-regional mobility and (b) too narrow to correctly identify locals from high- or low-Sr regions of Denmark. For example, an individual moving from a high-Sr region (e.g., 0.7115) to a low-Sr region (e.g., 0.7090) would be classified as "local" in both places, rendering the baseline ineffective for tracking mobility within Denmark.

We fully agree here and hope to have clarified this point with the comment above and with adding necessary clarifications in the text.

2. Misapplication of Statistical Methods

The statistical method chosen (Median Absolute Deviation, or MAD, to clean the data, followed by 2 sigma to define the range) is a valid approach for defining a local baseline at a single site, where it is reasonable to assume the majority of individuals are local to that site's specific geology.

However, applying this method to a composite national dataset drawn from hundreds of different sites with different geological backgrounds is statistically inappropriate. The resulting range (0.7089–0.7117) is not a "baseline" for mobility studies; it is merely a statistical summary of the central 95% of all bioavailable Sr values in Denmark.

The reviewer’s comment is rooted in the issues raised in the previous points. We would like to reiterate that the geological context of Denmark is rather unique: “basement” sediments are effectively shielded by the much more homogeneous glaciogenic tillites at the surface preventing the underlying geological sediments from significantly influencing the bioavailable strontium signal. Instead, bioavailable strontium is derived primarily from the overlying till deposits, and exhibits a relatively homogeneous isotope compositional distribution, as shown in the isoscape maps of Frei et al. (2022).

Based on this geological setting, it is reasonable to assume that most individuals are “local”—not to a specific archaeological site, but within the present-day borders of Denmark (as noted in our earlier response). Such an assumption would not be feasible in many other countries, where exposed and geologically complex substrates exert a more variable influence on bioavailable strontium. The Danish situation should therefore be regarded as a special case.

As mentioned previously, we also agree with the reviewer that the outlier-free range should not be referred to as a “baseline.” Instead, we use the term “human-based bioavailable strontium isotope range” for Denmark.

This method statistically defines individuals from the highest- and lowest-Sr regions of Denmark as "non-local," which is a critical error. This has direct implications for the case study: the six "non-local" individuals from Sølager may not be from outside Denmark; they may simply be from a part of Denmark with a naturally high-Sr signature that was statistically trimmed from the "local" range by the MAD analysis.

While in principle this is correct, high- and low Sr regions within Denmark with respect to relevant bioavailable strontium isotope signatures valid for past environmental conditions have not been identified thus far, despite the considerable amount of baseline samples that have been so far measured (for references on these please see Frei et al. 2022). This point is also discussed in the supplement material for convenience.

As noted above, we have now interpreted the non-elite Bronze Age individuals in relation to the two baselines currently available for provenance studies in Denmark (Frei & Frei 2011; Frei & Price 2012). In addition, we compare these interpretations with those derived from the human-based bioavailable strontium isotope range generated from the full dataset. We believe that presenting all three perspectives provides the reader with a more comprehensive understanding of how different reference materials may influence the interpretation of the archaeological samples.

3. Failure to Demonstrate Suitability (A Path Forward)

The authors criticize environmental isoscapes but fail to provide any evidence that their method is superior. To validate their claims, they must directly compare their "simple baseline" against a spatially-explicit model.

We want to emphasize that we are not criticizing environmental isoscapes as a concept. We do not mean to imply that the human-based bioavailable range is inherently superior to other methods. We recognize that, for provenance studies of the past, there is no “one-size-fits-all” approach. Our intention is to provide a comprehensive, updated, and easily accessible reference resource for the region of Denmark. We have clarified this point more explicitly in the revised manuscript.

Given that the 628 data points in the database are geolocated (as implied by Figure 1), the authors are in a prime position to do this. The critical revision required for this manuscript is as follows:

• The authors must use their 628 human data points to generate a human-derived 87Sr/86Sr isoscape for Denmark using appropriate spatial statistics (e.g., kriging).

We believe that human data (although the archaeological site locations are somewhat geolocated), due to the nature of information inherent to human samples, itself is less suitable as geolocated data, as compared to data from e.g. plant samples. This is because, the isotope signatures of the individuals are not constrained to particular areas/sites as we must assume that individuals also moved within Denmark. As correctly stated by the reviewer in above comments, the “local” and “non-local” criteria only refers to a country-wide isotope range of bioavailable strontium, but constructing an isoscape (f.e. via kriging) would , in our opinion, add potential errors due to the nature of the human data. Furthermore, such an isoscape would then not be comparable with any other baseline models for the reasons also mentioned above, as isoscapes based on modern archive proxies such as soil-leachates and plants likely do not reflect the distributions of bioavailable strontium in the past. We provide a detailed comparison of the statistically defined human-based bioavailable strontium isotope range with baselines and isoscapes constructed with modern environmental proxy archive data in the supplement, to elaborate on this point.

• They must then compare the predictive power of this new isoscape against their "simple baseline."

We no longer use the term “baseline” but refer to only a “range”.

• This re-analysis would allow them to re-evaluate the Sølager and Kalvehavegård individuals against a spatially-relevant local value (as predicted by their new human isoscape), rather than an arbitrary national average.

We now also compare the Sølager and Kalvehavegård data to the previous baselines published relevant for the past, providing a broader understanding that there are different ways in which the Sølager and Kalvehavegaard data can be interpreted.

Without this spatial analysis, the paper's central argument is unsubstantiated, and its conclusions regarding non-elite mobility are unreliable.

As mentioned above, we have now re-evaluated and re-interpreted the non-elite Bronze Age individuals applying various reference frameworks.

As this is the first study to investigate non-elite individuals from the Nordic Bronze Age using strontium isotope analysis, we consider it a valuable contribution to the archaeological community. Furthermore, the comprehensive dataset presented here—including 81 previously unpublished samples from western Jutland, a region typically characterized by poor skeletal preservation—provides, for the first time, a large number of human strontium isotope data across this underrepresented area.

Conclusion

The dataset presented is of great value to the archaeological community. However, the methodology used to interpret this data is flawed and ignores the foundational principles of strontium provenance. The "simple baseline" approach is inappropriate for a nationwide study.

I recommend Major Revisions. The paper cannot be accepted until the authors address this fundamental methodological flaw, preferably by conducting the spatial analysis suggested above and re-evaluating their conclusions accordingly.

We hope that our revised manuscript, including the clarifications and our re-evaluations of the conclusions, will be considered suitable for publication. While we acknowledge the reviewer’s suggestion to conduct a spatial analysis of the human dataset, we hope that our reasoning has clarified why we have chosen to refrain from doing so in this study. At the same time, we fully agree with the reviewer’s point regarding terminology and have therefore discontinued the use of the term “baseline” for our reference material, instead referring to it as a “human-based bioavailable strontium isotope range”.

6. PLOS authors have the option to publish the peer review history of their article (what does this mean?). If published, this will include your full peer review and any attached files.

If you choose “no”, your identity will remain anonymous but your review may still be made public.

Do you want your identity to be public for this peer review? For information about this choice, including consent withdrawal, please see our Privacy Policy.

Reviewer #1: No

Reviewer #2: No

________________________________________

[NOTE: If reviewer comments were submitted as an attachment file, they will be attached to this email and accessible via the submission site. Please log into your account, locate the manuscript record, and check for the action link "View Attachments". If this link does not appear, there are no attachment files.]

To ensure your figures meet our technical requirements, please review our figure guidelines: https://journals.plos.org/plosone/s/figures

You may also use PLOS’s free figure tool, NAAS, to help you prepare publication quality figures: https://journals.plos.org/plosone/s/figures#loc-tools-for-figure-preparation.

NAAS will assess whether your figures meet our technical requirements by comparing each figure against our figure specifications.
